# Influence of scleral thickness on photodynamic therapy outcomes in central serous chorioretinopathy

**DOI:** 10.1111/aos.16779

**Published:** 2024-10-20

**Authors:** Paolo Forte, Jennifer Cattaneo, Felice Cardillo Piccolino, Alessandro Arrigo, Paolo Corazza, Donatella Musetti, Raffaella Rosa, Carlo Enrico Traverso, Vincenzo Fontana, Marco Lupidi, Chiara Maria Eandi, Massimo Nicolò

**Affiliations:** ^1^ Eye Unit IRCCS Ospedale Policlinico San Martino Genoa Italy; ^2^ DINOGMI University of Genoa Genoa Italy; ^3^ Department of Ophthalmology, Jules‐Gonin Eye Hospital, Fondation Asile des Aveugles University of Lausanne Lausanne Switzerland; ^4^ Fondazione Italiana Macula ETS Genoa Italy; ^5^ Department of Ophthalmology IRCCS San Raffaele Scientific Institute Milan Italy; ^6^ Clinical Epidemiology Unit IRCCS Ospedale Policlinico San Martino Genoa Italy; ^7^ Eye Clinic, Department of Experimental and Clinical Medicine Polytechnic University of Marche Ancona Italy; ^8^ Department of Surgical Sciences University of Torino Torino Italy

**Keywords:** anterior segment optical coherence tomography, central serous chorioretinopathy, choroidal thickness, photodynamic therapy, scleral substantia propria, scleral thickness

## Abstract

**Purpose:**

To test the prognostic role of anterior scleral substantia propria (ASSP) thickness in predicting the 3‐month response after half‐dose photodynamic therapy (PDT) in central serous chorioretinopathy (CSCR) and to assess its clinical relevance of ASSP in different CSCR phenotypes.

**Methods:**

A prospective, exploratory, multi‐centre cohort study conducted at IRCCS San Martino Hospital (Genoa, Italy) and Jules‐Gonin Eye Hospital (Lausanne, Switzerland). Demographic and clinical data, and optical coherence tomography (OCT) were collected at baseline and 3 months after PDT. Based on OCT images, we categorized CSCR phenotypes and collected clinically relevant imaging metrics. ASSP thickness was obtained from four different measurements using anterior segment (AS) OCT. Multivariable regression models were performed to evaluate the distribution of ASSP thicknesses among different CSCR phenotypes and to test the prognostic role of ASSP thickness in discriminating between PDT responders (complete subretinal fluid reabsorption) and partial responders.

**Results:**

The study cohort comprised 109 Caucasian patients (82 males, 75.2%) with a total of 142 eyes: 84 eyes *simple* (59.1%) versus 58 eyes *complex* (40.9%) CSCR. A linear normal model confirmed a positive association between *complex* CSCR and higher ASSP thickness (*β* = 26.1, 95% CL = 12.1/40.1, *p* < 0.001), with a low prevalence of ciliochoroidal effusion loculations in AS‐OCT (1/142 eyes, 0.7%). ASSP thickening was positively linked to the presence of posterior cystoid retinal degeneration (PCRD; *p* = 0.002), indicating a potential role in the pathogenesis of severe CSCR phenotypes. In the subgroup of treated patients (61 eyes), 63.9% had a complete response after PDT. In these patients a logistic binary model highlighted a significantly higher risk of PDT non‐responsiveness (OR = 9.62, 95% CL = 2.44/37.9, *p* = 0.001) associated with a 60‐unit increase in ASSP thickness levels. By contrast, other anatomical parameters (i.e., body surface area, age, gender, axial length) showed no remarkable prognostic roles.

**Conclusion:**

This research highlighted the association of ASSP thickening with *complex* CSCR phenotype in Caucasian patients and its role in predicting PDT efficacy. These findings enhance our comprehension of the anatomical risk factors in patients affected with CSCR and potentially guide a better understanding of non‐responsive cases to PDT treatment.

## 1 INTRODUCTION

Central serous chorioretinopathy (CSCR) is a chorioretinal disease characterized by an idiopathic neurosensory retinal detachment at the posterior pole, often associated with focal retinal pigment epithelium (RPE) detachments (Gass, 1967). The identification of multiple risk factors associated with CSCR has led to the identification of three main pathogenic pathways in the disease: (1) a condition of increased ocular perfusion pressure (OPP) (Cardillo Piccolino et al., 2018a, 2018b; Lupidi et al., 2020) where risk factors are arterial hypertension (Tittl et al., 1999), low intraocular pressure (IOP) (Cardillo Piccolino, 1981), increased blood pressure variability (Karadağ, 2021), and systemic hemodynamic changes favoured by peculiar psychosomatic aspects (Spahn et al., 2003; Yannuzzi, 1986) or by vigorous physical activity (Cardillo Piccolino et al., 2022), associated with an ineffective choroidal vascular reactivity (Lupidi et al., 2020; Polska et al., 2007; Tittl et al., 2005); (2) an impaired choroidal response to corticosteroid drug administration (Haimovici et al., 2004) and an abnormal ocular endogenous corticoid metabolism (Zola et al., 2023); (3) a peculiar anatomical risk profile linked to short axial length (AXL) (Oh et al., 2014; Terao et al., 2020), choroidal folds (Cohen et al., 2022), and thickened chorioscleral compartment (Imanaga et al., 2023); in particular this subgroup of risk factors could account for the interocular asymmetry of CSCR manifestations.

The involvement of scleral substantia propria, also known as the scleral stroma (Boote et al., 2020), in the pathogenesis of CSCR was initially postulated by Gass in 1983 (Gass, 1983). Since the intricate relationship between choroidal and scleral thickness and its actual contribution to the disease pathogenesis has not been fully elucidated, our aim was to focus on this particular anatomical risk factor and confirm its implication specifically in Caucasian patients.

Recent investigations covering the measurement of anterior scleral substantia propria (ASSP) thickness were based on anterior segment optical coherence tomography (AS‐OCT). These studies specifically assessed the ASSP thickness at a point situated 6 mm posteriorly to the scleral spur (SS), as the vertical distance between the chorioscleral interface and the corresponding hyporeflective recti muscles (RM) body or perimysia (Imanaga et al., 2021). With these reproducible reference landmarks, previous authors demonstrated a significantly greater ASSP thickness in CSCR patients compared to healthy controls (Fernández‐Vigo et al., 2021; Lee et al., 2021). Additionally, a notable finding includes a significantly thinner ASSP thickness in cases of steroid‐induced CSCR when compared to idiopathic onset (Sawaguchi et al., 2022). It is important to note that this imaging technique offers an axial resolution of ~10 μm (Imanaga et al., 2021), which overcomes the resolution of contact B‐scan ultrasonography, with approximately 150 μm (Silverman, 2009) and 80 μm (Spaide et al., 2022) for 10 and 20 MHz probes, respectively.

Since ultra‐widefield indocyanine green angiography (ICGA) studies have demonstrated an asymmetrical vortex vein (VV) drainage (Hiroe & Kishi, 2018), inter‐VV anastomoses (Matsumoto et al., 2020), and an overall congestion of venous choroidal outflow in CSCR patients (Pauleikhoff et al., 2023), the implication of a thick sclera directly compressing the intrascleral VV tract has been hypothesized as the causative factor for reduced choroidal outflow and engorged VV ampullae (Spaide, 2020). Beyond influencing the distribution of choroidal venous outflow, a thick and rigid sclera may also predispose a diminished transscleral bulk protein outflow—a well‐established mechanism in studies covering uveal effusion syndrome (UES) (Johnson, 1999)—leading to fluid loculations in posterior choroid (Spaide & Ryan, 2015) and in the suprachoroidal space (Imanaga et al., 2021).

We designed a prospective study with the aim to investigate the potential prognostic significance of ASSP thickness in predicting a complete subretinal fluid (SRF) resolution at 3‐month follow‐up after ICGA‐guided half‐dose verteporfin photodynamic therapy (PDT) (Nicoló et al., 2014). We also wanted to stratify the thickness of the ASSP in different CSCR imaging‐based phenotypes in Caucasian patients, specifically distinguishing between *simple* and *complex* manifestations, according to the multimodal imaging‐based classification system proposed by the CSCR International Study Group (Chhablani et al., 2020).

## 2 METHODS

### 2.1 Study design and population

In this prospective, exploratory, multi‐centre, cohort study we collected the clinical records of consecutive patients diagnosed with CSCR at baseline and, when applicable, at 3‐month follow‐up after ICGA‐guided half‐dose PDT. Recruitment of patients occurred between January 2023 and January 2024 from two academic retinal centres, specifically the University Eye Clinic DINOGMI at Policlinico San Martino IRCCS Hospital (University of Genoa, Italy) and Jules‐Gonin Eye Hospital (University of Lausanne, Switzerland). The study was approved by the cantonal commission for ethics and human research committee (CER‐VD 2017‐00493) and was performed in agreement with the principles outlined in the Declaration of Helsinki for research involving human subjects. The initial diagnosis of CSCR was established based on the presence of serous macular detachment associated with pachychoroid detected with OCT, RPE focal leakage and choroidal vascular hyperpermeability detected with fluorescein angiography (FA) and ICGA, respectively (Cardillo Piccolino et al., 1995). Patients with the following conditions were excluded from the study: (1) any other chorioretinal or optic nerve disorder; (2) relevant optic media opacities and/or insufficient fixation to allow high‐quality imaging; (3) any history or detection of scleritis or episcleritis; (4) the inability to measure ASSP thickness due to sub‐optimal image quality; (5) ongoing childbearing or history of pregnancy in the previous 12 months; (6) history of glaucoma filter surgery or ocular hypotony (<8 mm Hg); (7) PDT or subthreshold laser treatments performed in the previous 12 months.

### 2.2 Data collection

Clinical records and multimodal imaging analysis were performed by independent blinded ophthalmologists (PF and JC). Comprehensive data acquisition included demographic features, laterality, previous treatments, medical and history of steroid drug exposure. All enrolled patients underwent a multimodal imaging evaluation that included early treatment diabetic retinopathy study (ETDRS) best correct visual acuity (BCVA), applanation tonometry (Goldman), AXL measurement (OA‐2000, Tomey, Nagoya, Japan), structural OCT (Spectralis HRA + OCT; Heidelberg Engineering, Heidelberg, Germany; protocol: volumetric 30° × 20° raster, 97 sections, 1530 A‐scans, high resolution, and enhanced depth imaging), FA and ICGA (Optos California, Optos, Dunferline, UK), colour fundus photography (CPF) and OCT‐Angiography (OCT‐A) (swept‐source DRI OCT Triton; Topcon Corporation, Tokyo, Japan; protocol: 4.5 mm × 4.5 mm cube centred at the fovea). Measurement of ASSP thickness was obtained in the four horizontal and vertical gaze positions (superior, temporal, inferior, nasal) using a scanning laser ophthalmoscope (Spectralis HRA + OCT; Heidelberg Engineering, Heidelberg, Germany). The following parameters were employed for the imaging protocol: linear 20° scan (1024 A‐scans), enhanced depth imaging and enhanced contrast prior to image analysis.

### 2.3 CSCR grading

The cohort was divided into two distinct subgroups, namely *simple* or *complex*, utilizing the classification system proposed by the CSCR International Group (Chhablani et al., 2020), grading both eyes at the same time (Chhablani et al., 2022). This classification was established based on the presence of RPE alterations spanning more than two‐disc diameters and/or multifocal RPE changes on en‐face imaging modalities, including fundus autofluorescence (FAF). Based on previous records about the clinical course, additional subgroups were classified: acute disease (patients experiencing the first reported episode of CSCR), recurrent disease (presence of SRF with either a history or signs of resolved episodes), resolved disease (absence of SRF with either history of signs of resolved episodes), and persistent disease (continuous duration of SRF for more than 6 months) (Chhablani et al., 2022; Daruich et al., 2015).

### 2.4 OCT grading

Baseline OCT images were first reviewed for eligibility by independent readers (PF and JC). The same readers, who were blind to the visual results, then conducted independent grading on each eligible eye for both qualitative and quantitative features. The mean value was employed for statistical analysis of metric features. For categorical features, in case of disagreement on a single result, a further assessment was performed by a third author (CME).

**FIGURE 1:**
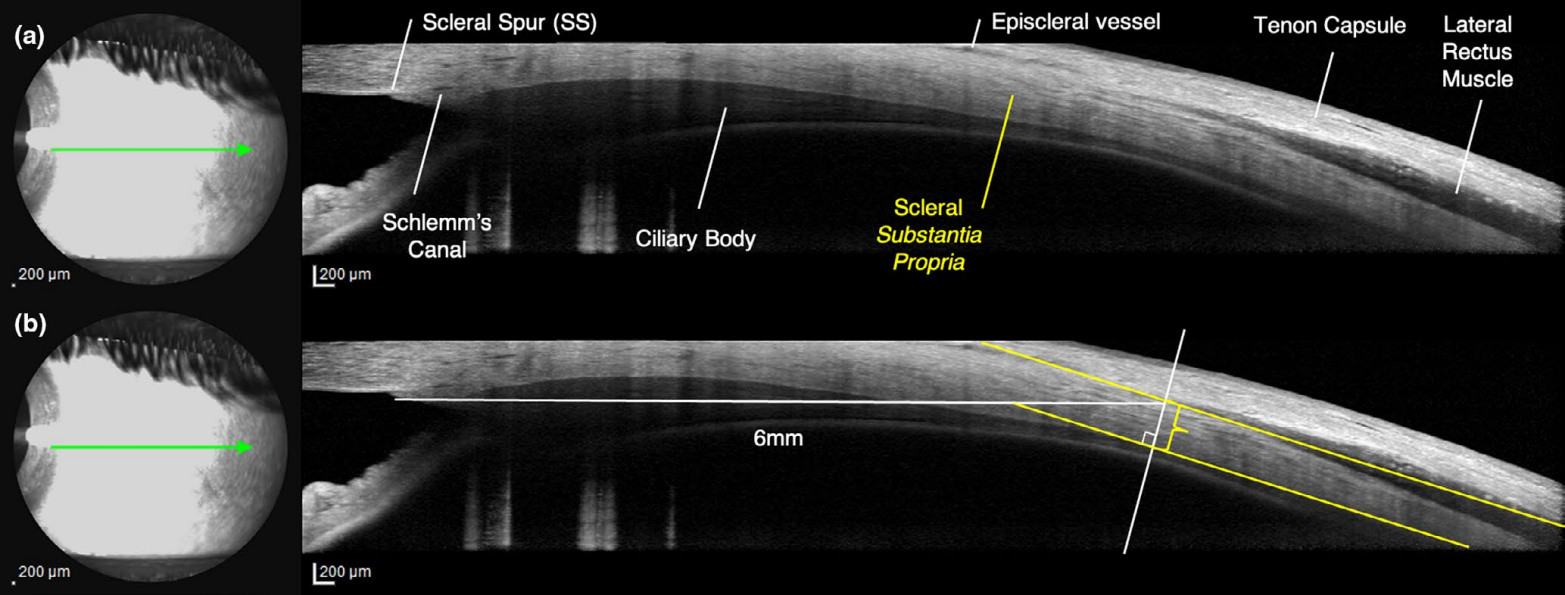
The measurement of anterior scleral substantia propria (ASSP) thickness was conducted in the four horizontal and vertical gaze meridians (superior, temporal, inferior, nasal) using a scanning laser ophthalmoscope. (a) Key landmarks included the scleral spur (SS), episcleral vessels (EV) characterized by posterior signal attenuation, and the recti muscles (RM) seen as low reflective bands. (b) The ASSP thickness (depicted by the yellow *brace*) was specifically assessed at a point located 6 mm posterior to the SS. This involved manually measuring the vertical distance between the chorioscleral interface and the corresponding hyporeflective recti muscles' body or perimysia (depicted by the yellow line drawn perpendicularly to the inner wall of the scleral stroma), as previously described by Imanaga et al. (2021).

The following baseline categorical OCT biomarkers were graded: serous pigment epithelium detachment (PED) (either single or multiple), flat irregular PED (FI‐PED) (Hwang et al., 2020) with or without detectable Type 1 macular neovascularization (presence of neovessels was confirmed with OCT‐A in analogy to Lupidi et al. (2020)), choroidal folds (CF), posterior cystoid retinal degeneration (PCRD) (Cardillo Piccolino et al., 2008), polypoidal vascular dilations (Quaranta‐El Maftouhi et al., 2015), and anterior fluid loculations on AS‐OCT. Additionally, subfoveal choroidal thickness (ChT) was measured manually using the OCT calliper measurement tool as the distance between Bruch's membrane and the scleral–choroidal interface. For the measurement of ASSP thickness, we referred to methods described in previous studies (Buckhurst et al., 2015; Imanaga et al., 2021). In particular, after the identification of the SS and the four RM as hyporeflective bands adjacent to the anterior sclera (Suzuki et al., 2018), ASSP thickness was manually measured 6 mm posteriorly to the SS as the vertical distance between the chorio‐scleral interface and the corresponding hyporeflective RM body or perimysia at four locations after identification of each rectus muscle, as previously described by Imanaga et al. (2021) (Figure 1).

### 2.5 Half‐dose PDT and endpoint

After drug‐induced mydriasis and local anaesthetic administration, an intravenous infusion of 3 mg/m^2^ (half‐dose) verteporfin (Visudyne; Novartis, Basel, Switzerland) was administered within 10 min. Body surface area (BSA) was estimated from weight (kg) and height (m) using the Mosteller formula (Mosteller, 1987). For a duration of 83 s, PDT laser at standard fluency (50 J/cm^2^) and at a wavelength of 689 nm was applied to non‐overlapping circular areas of 1100 μm of diameter, chosen based on the hyperfluorescent abnormalities on middle phase ICGA (Yannuzzi et al., 2003). All procedures were performed by a single operator (MN). The endpoint to binarily define treatment efficacy was a complete resolution of SRF (both macular and extramacular) on OCT at 3‐month follow‐up after a single half‐dose (HD) PDT session. In cases of SRF associated with Type 1 MNV, HD‐PDT was performed as a standalone treatment and was not associated with anti‐VEGF injections. Visits performed within 2 weeks from the scheduled timeframe were considered acceptable for analysis.

### 2.6 Statistical analysis

The absolute and relative (%) frequency distribution of each categorical clinical finding was explored using the analysis of contingency tables and the chi‐squared test was applied to assess differences between *simple* and *complex* cases. Metric biomarkers were described using the median and the interquartile range (IQR), and the Mood's median test was performed to compare the biomarker distributions in the two CSCR groups.

The two main endpoints of our clinical investigation were addressed using two distinct multivariable regression methods: (1) a linear normal model (LNM) for analysing the associations of ASSP thickness with CSCR phenotype and SRF features; (2) a logistic binary model (LBM) for estimating the prognostic effect of ASSP thickness on a complete SRF response in patients treated with verteporfin PDT at 3‐month follow‐up. Regression coefficients were directly used as indexes of association in LNM analysis whereas odds ratios (OR) were computed in LBM context. The covariates considered in both regression settings were selected according to a critical review of previous literature on CSCR topic and integrated with the salient features specific to scleral biomechanical properties. Given that clinical measurements carried out on paired organs are in general positively correlated, to reduce the impact of inter‐eye correlation on statistical inference, sampling variabilities of all regression parameters and related 95% confidence limits (95% CL) were computed using a generalized estimating equation approach (Ying et al., 2021). A two‐sided *p*‐value ≤0.05 was considered as statistically significant. In addition, the Pearson correlation test has been used to assess the statistical relationship among the collected variables. All data analyses were conducted using Stata Statistical Software: Release 17 (StataCorp. 2021).

## 3 RESULTS

### 3.1 Descriptive analysis

The study cohort comprised 109 Caucasian patients (male: 82, 75.2%) with a total of 142 eyes. Among the study population, 33 patients (30.3%) contributed with both eyes while 76 patients (69.7%) with one eye. At baseline, 105 eyes displayed pachyvessels (73.9%), 38 PED (26.7%), 22 multiple PED (15.4%), 27 FI‐PED (19.0%) of which 16 with detectable Type 1 MNV on OCT‐A (11.3%), 12 PCRD (8.4%), 8 polypoidal vascular dilations (5.6%) and 4 choroidal folds (2.8%). Mean IOP was 14.4 ± 3.6 mmHg. SRF was acute in 17 eyes (15.6%), persistent in 36 eyes (25.3%), recurrent in 41 eyes (28.9%), resolved in 38 eyes (26.7%) and steroid‐related in 11 eyes (7.7%).

Tables 1 and 2 present, respectively, the distribution of metric and categorical biomarkers in relation to the CSCR classification: 84 eyes *simple* (59.1%) versus 58 eyes *complex* (40.9%).

**TABLE 1:** Distribution of metric recordings according to CSCR clinical classification.

Variable		*Simple*		*Complex*		*p*‐value
		Median	IQR	Median	IQR	
Age (years)		51.5	41.5/58.0	50.5	42.0/56.0	0.523
Axial length (mm)		23.4	22.8/24.3	23.1	22.6/23.6	0.162
BCVA (ETRS)		80.0	74.0/85.0	77.0	66.0/85.0	0.475
Subfoveal ChT (μm)		421.0	335.0/471.0	489.5	422.0/536.0	<0.001*
Maximal ChT (μm)		444.5	341.0/499.5	503.0	450.0/545.0	0.003*
ASSP thickness (μm)	Temporal	425.5	400.5/468.5	483.5	442.0/526.0	0.001*
	Inferior	440.0	405.0/468.5	463.0	428.0/490.0	0.012*
	Nasal	430.0	405.0/462.5	447.0	413.0/498.0	0.026*
	Superior	414.0	386.0/445.0	456.5	426.0/480.0	<0.001*
	Mean	432.0	405.3/456.6	460.3	434.8/493.5	<0.001*
Total		84 eyes (59.1%)		58 eyes (40.9%)		

Abbreviations: ASSP, anterior scleral substantia propria; BCVA, best correct visual acuity (ETDRS); ChT, choroidal thickness; IQR, interquartile range; *p*‐value, probability level associated with the nonparametric median test; *, statistical significance.

**TABLE 2:** Distribution of categorical variables according to CSCR classification.

Variable		*Simple*		*Complex*		Total		*p*‐value
		*N*	%	*N*	%	*N*	%	
Male gender		60	55.8	48	44.4	108	76.0	0.120
PED		33	55.0	27	45.0	60	42.3	0.341
Multiple PED		12	54.6	10	45.5	22	15.5	0.635
FIPED with Type 1 MNV (OCT‐A)		8	50.0	8	50.0	16	11.3	0.429
PCRD		1	8.3	11	91.7	12	8.5	<0.001*
Choroidal folds		0	0.0	4	100.0	4	2.8	0.015*
CSCR episode	Acute (<6 m)	16	94.1	1	5.9	17	12.0	0.002*
	Persistent (>6 m)	7	19.4	29	80.6	36	25.4	<0.001*
	Recurrent	28	68.3	13	31.7	41	28.9	0.158
	Resolved	27	71.1	11	28.9	38	26.8	0.081
	Steroid‐related	7	63.6	4	36.3	11	7.7	0.753
Total		84	59.1	58	40.9	142	100.0	–

Abbreviations: FI‐PED, flat irregular PED; MNV, macular neovascularization; *N*/%, absolute/relative frequency; OCT‐A, optical coherence tomography angiography; PCRD, posterior cystoid retinal degeneration; PED, pigment epithelium detachment; *p*‐value, probability level associated with the one‐degree‐of‐freedom chi‐square test; *, statistical significance.

Age (*p*‐value = 0.523), AXL (*p*‐value = 0.162) and BCVA (*p*‐value = 0.475) did not show significant differences between the two groups, while subfoveal ChT measurements and regional variations in ASSP thickness appear to be relevant factors in distinguishing between *simple* and *complex* CSCR cases (mean values: 432 and 460 μm, respectively, *p*‐value < 0.001).

Categorical variables highly represented in the *complex* group were the presence of PCRD (12 vs. 1 case) and choroidal folds (4 vs. 0 cases) both demonstrating significant imbalances (*p*‐value < 0.001 and *p*‐value = 0.015, respectively). On the contrary, acute SRF (12% of total cases) was more represented in the *simple* group (94.1% vs. 5.9%, *p*‐value = 0.002) while, persistent SRF (25.4% of total cases) was more common in the *complex* group (19.4% vs. 80.6%, *p*‐value < 0.001).

### 3.2 Linear regression modelling

**FIGURE 2:**
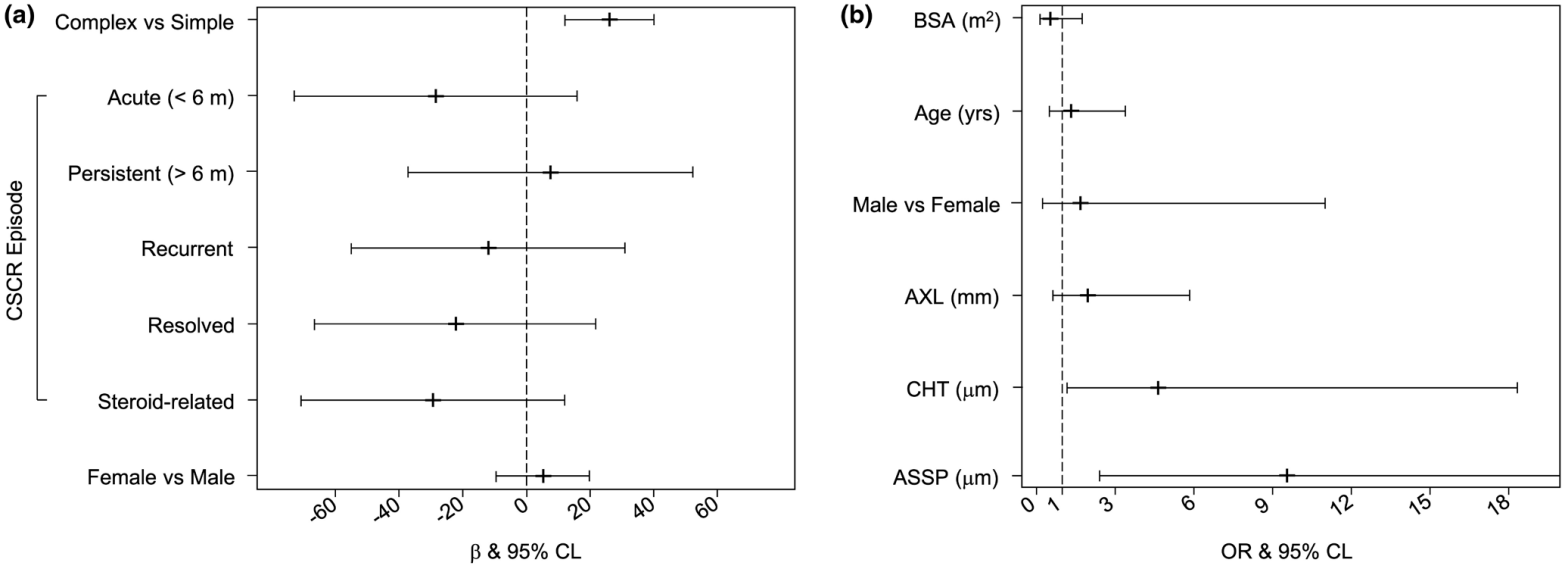
(a) Caterpillar plot for the linear normal model (LNM) analysing the associations of anterior scleral substantia propria (ASSP) thickness with central serous chorioretinopathy (CSCR) imaging phenotype and episode; (b) Caterpillar plot for the logistic binary model (LBM) for estimating the prognostic effect of ASSP thickness on an incomplete SRF response in patients treated with verteporfin HD‐PDT at 3 months.

As displayed in Table 3, the LNM indicated a significant positive association between *complex* CSCR and greater ASSP thickness (*β* = 26.1, 95% CL = 12.1/40.1) while remarkable lower ASSP thickness values were found in case of acute SRF (*β* = −28.6, 95% CL = −73.0/15.8) and steroid‐related SRF (*β* = −29.5, 95% CL = −70.9/11.9), as displayed in Figure 2a. In addition, considering the CSC‐related collected variables, ASSP thickness resulted significantly correlated with PCRD (Pearson correlation coeff. = 0.262; *p*‐value = 0.002).

**TABLE 3:** Multivariable linear normal regression modelling of ASSP thickness based on CSCR clinical phenotypes and classification.

Variable		*β*	95% CL	*p*‐value
Classification	*Complex* vs. simple	26.1	12.1/40.1	<0.001*
CSCR episode	Acute (<6 m)	−28.6	−73.0/15.8	0.207
	Persistent (>6 m)	7.50	−37.1/52.1	0.742
	Recurrent	−12.0	−55.1/31.0	0.584
	Resolved	−22.3	−66.5/21.9	0.323
	Steroid‐related	−29.5	−70.9/11.9	0.163
Gender (female vs. male)		5.0	−9.6/19.7	0.501
Constant		456.8	413.5/5002	–

*Note*: A generalized estimating equation (GEE) approach was used to control for the within‐patient correlation (142 eyes of 109 patients).

Abbreviations: 95% CL, 95% confidence limits for *β*; *p*‐value, probability level associated with the Wald test; *β*, regression coefficient (mean difference) adjusted for axial length (AXL), age gender and collaborative centre; *, statistical significance.

### 3.3 PDT response subgroup

Sixty‐one eyes of 56 patients completed the 3‐month follow‐up after PDT. In 39/61 eyes (63.9%), SRF was completely reabsorbed (PDT responders), while in 22/61 eyes (36.1%) response was incomplete (PDT partial responders). Table 4 presents the results of LBM analysis carried out on PDT as a binary indicator (partial response vs. response) using anatomical parameters, evaluated immediately prior to treatment, as prognostic predictors. Interestingly, the prognostic effect of most of the anatomical factors remained practically the same across the two PDT groups while ChT and ASSP thickness showed significant excesses of risk of partial response (Figure 2b). In particular, using the IQR of both predictors as measurement unit for risk quantification, increments of about 5 times (OR = 4.68, IQR = 142 μm, 95% CL = 1.19/18.5, *p*‐value = 0.027) and about 10 times (OR = 9.62, IQR = 60 μm, 95% CL = 2.44/37.9, *p*‐value = 0.001) were estimated for ChT and ASSP thickness, respectively.

**TABLE 4:** Logistic multivariable regression modelling of PDT non‐responders based on patient anatomical parameters evaluated immediately prior to treatment.

Variable	OR	95% CL	*p*‐value
BSA—linear trend per IQR = 0.03‐m^2^ increase	0.52	0.15/1.76	0.291
AGE—linear trend per IQR = 4‐year increase	1.31	0.50/3.42	0.579
GENDER—male vs. female	1.66	0.24/11.1	0.602
AXL—linear trend per IQR = 1.7‐mm increase	1.96	0.65/5.92	0.234
CHT—linear trend per IQR = 142‐μm increase	4.68	1.19/18.5	0.027*
ASSP thickness (mean)—Linear trend per IQR = 60‐μm increase	9.62	2.44/37.9	0.001*

*Note*: A generalized estimating equation (GEE) approach was used to control for the within‐patient correlation (61 eyes of 56 patients).

Abbreviations: 95% CL, 95% confidence limits for OR; ASSP, anterior scleral substantia propria; AXL, axial length; BSA, body surface area; CHT, subfoveal choroidal thickness; IQR, interquartile range; OR, odds ratio; *p*‐value, probability level associated with the Wald test; *, statistical significance.

## 4 DISCUSSION

**FIGURE 3:**
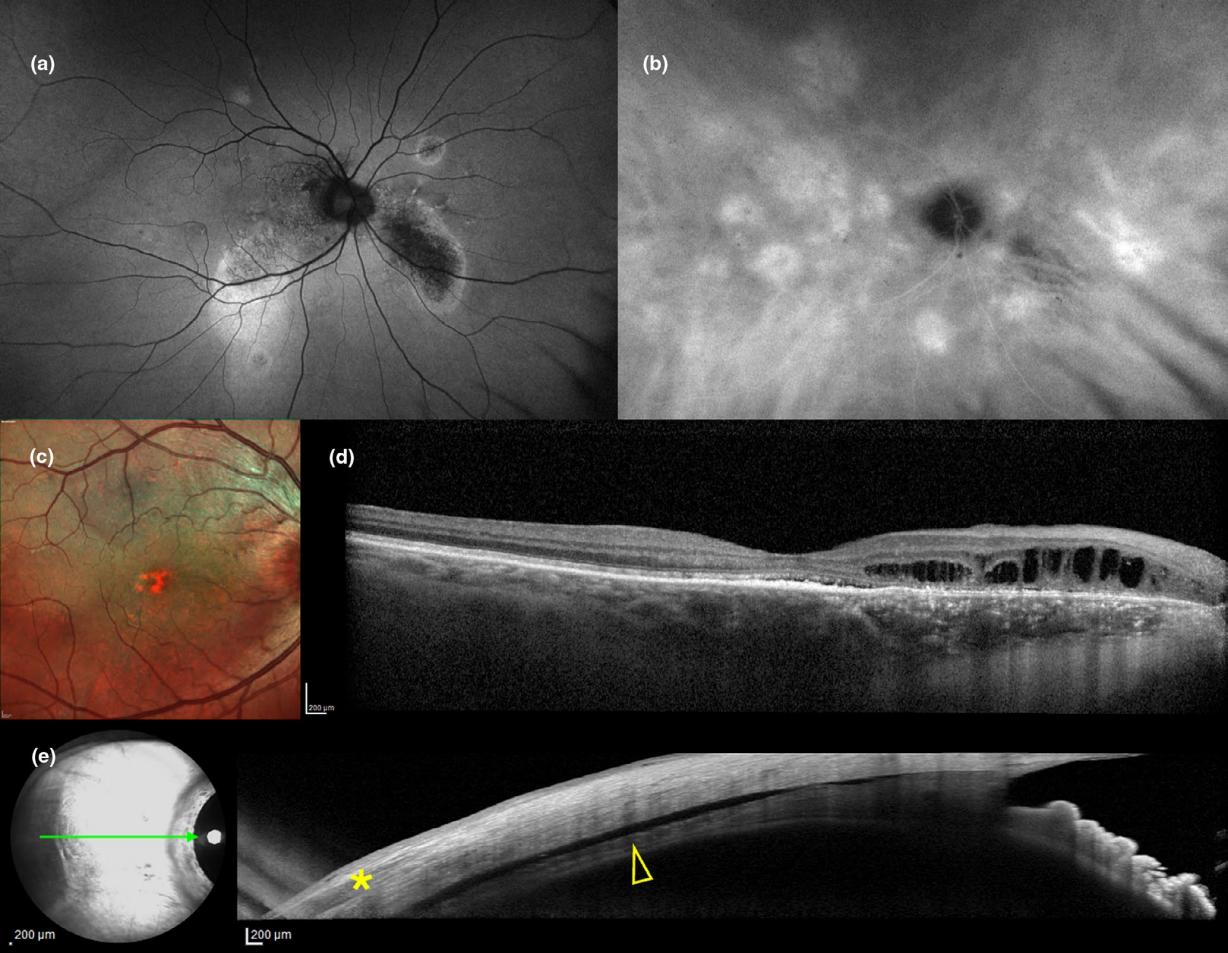
Multimodal imaging evaluation including anterior scleral substantia propria (ASSP) in a male patient (60‐years old) affected by *complex* central serous chorioretinopathy (CSCR) and *persistent* subretinal fluid (SRF). (a) Fundus autofluorescence reveals the presence of retinal pigment epithelium (RPE) alterations spanning more than two‐disc diameters, as well as discrete areas of hypoautofluorescence corresponding to RPE atrophy. (b) Late‐frames (20:00 min) indocyanine green angiography (ICGA) shows widespread areas of choroidal vascular hyperpermeability. (c) Multicolor imaging and (d) corresponding horizontal optical coherence tomography (OCT) scan display a nasal area of posterior cystoid retinal degeneration (PCRD) associated with choroidal thickening and posterior fluid accumulation. (f) With the eye positioned in nasal gaze, temporal scleral layers are exposed enabling anterior segment (AS) OCT imaging of ASSP thickness, 6 mm posteriorly to the scleral spur (SS). Note the presence of the hyporeflective lateral rectus muscle (*asterisk*) and the anterior accumulation of fluid in the suprachoroidal space (i.e., ciliochoroidal effusion; *arrowhead*), in the context of a diffuse ASSP thickening. Axial length (AXL) from the study eye was 22.65 mm.

**FIGURE 4:**
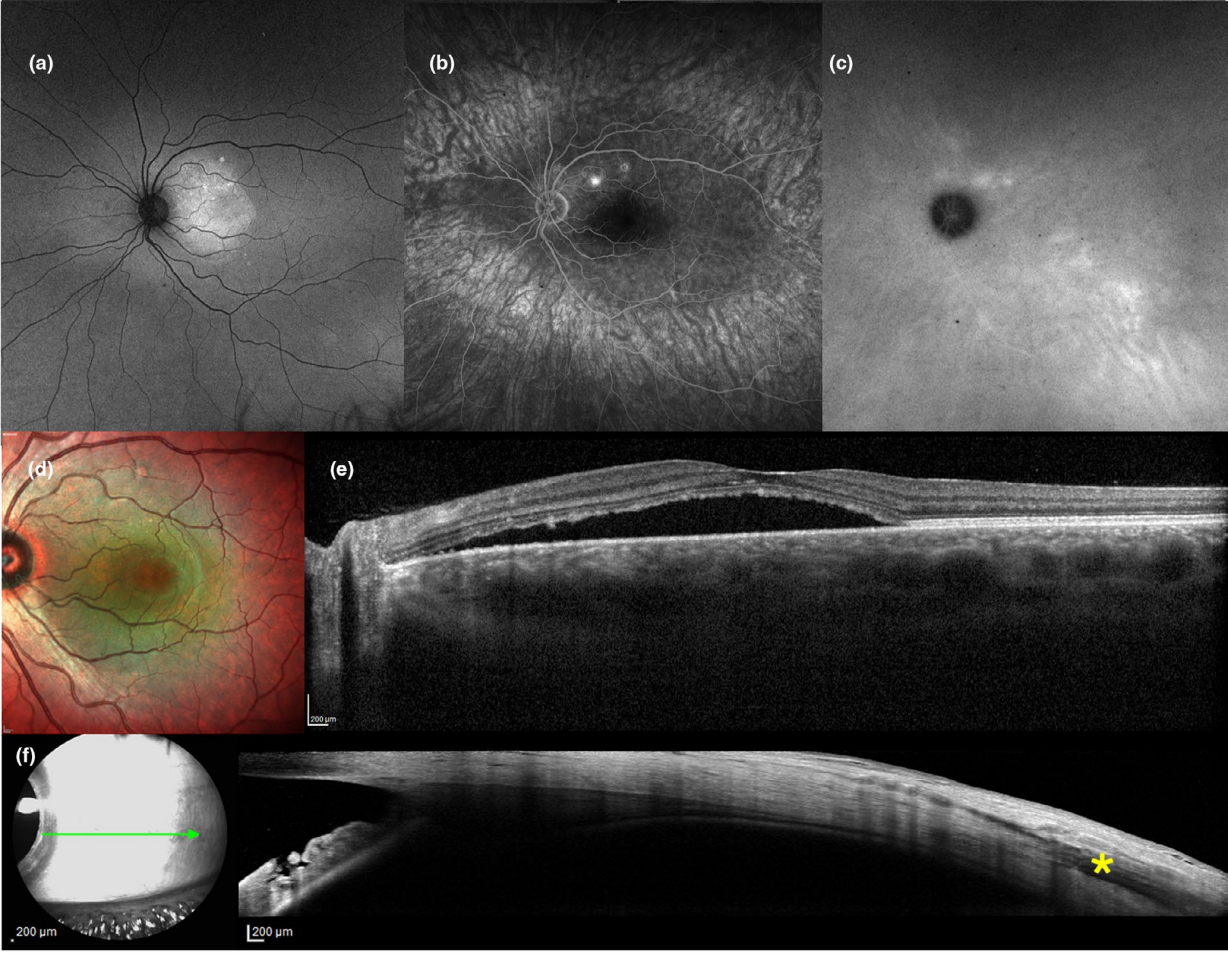
Multimodal imaging evaluation including anterior scleral substantia propria (ASSP) in a male patient (46‐years old) affected by *simple* central serous chorioretinopathy (CSCR) and *acute* subretinal fluid (SRF). (a) Fundus autofluorescence reveals the presence of retinal pigment epithelium (RPE) alterations spanning less than two‐disc diameters, and the presence of a hyperautofluorescent neuroretinal elevation. (b) Late‐frames (10:00 min) fluorescein angiography (FA) shows the presence of a focal leaking point along the superotemporal macular arcade. (c) Late frames (20:00 min) indocyanine green angiography (ICGA) shows discrete areas of choroidal vascular hyperpermeability. (d) Multicolor imaging and (e) corresponding horizontal optical coherence tomography (OCT) scan display a serous neuroretinal detachment associated with choroidal thickening and posterior fluid accumulation. (e) Anterior segment (AS) OCT imaging of ASSP thickness, 6 mm posteriorly to the scleral spur (SS). Note the presence of the hyporeflective lateral rectus muscle (*asterisk*) and the backshadowing effect by the episcleral vessels (EV), which provide an additional landmark to localize the interface between episcleral tissue and scleral substantia propria. Axial length (AXL) from the displayed eye was 23.80 mm.

**FIGURE 5:**
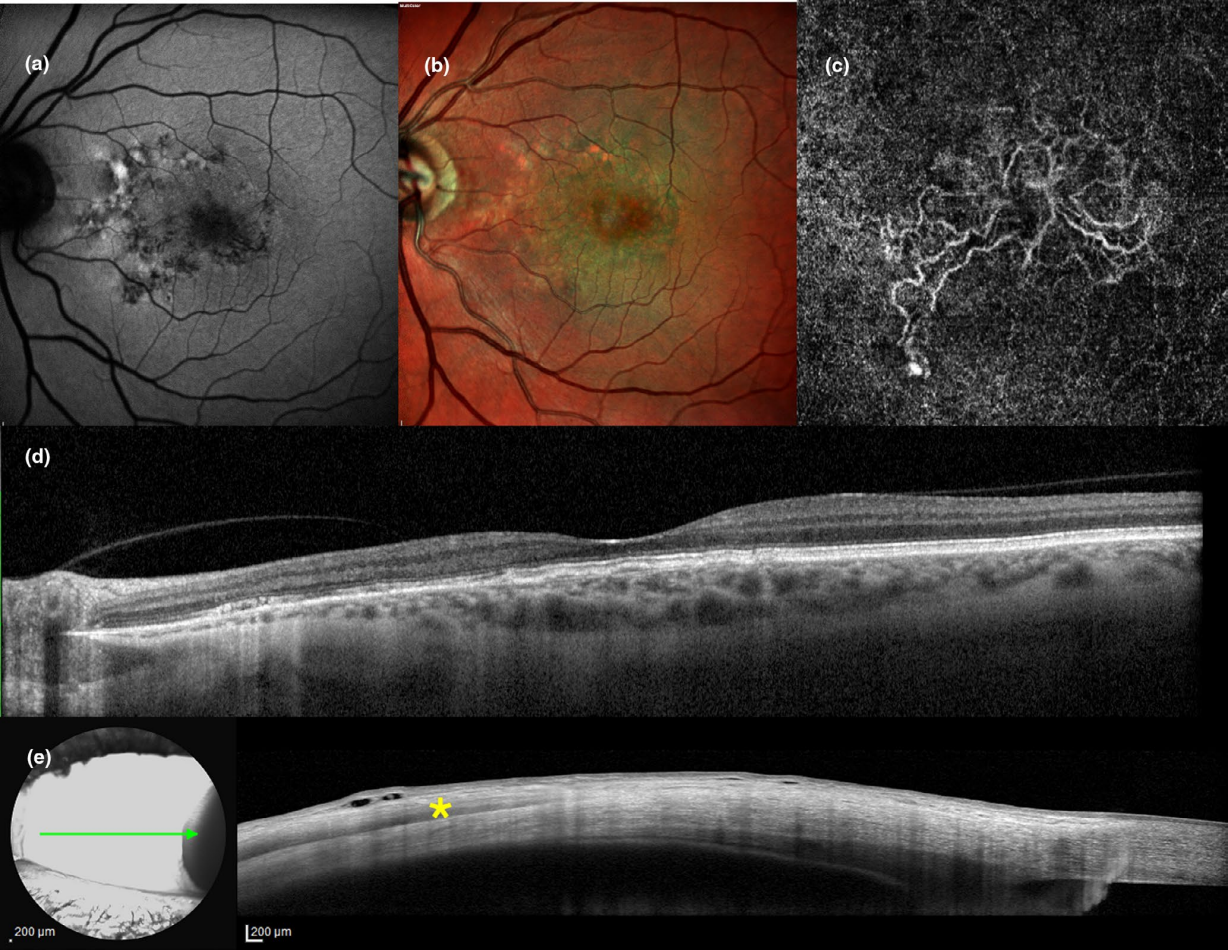
Multimodal imaging evaluation including anterior scleral substantia propria (ASSP) in a female patient (53‐years old) affected by *steroid‐induced* central serous chorioretinopathy (CSCR). (a) Fundus autofluorescence reveals the presence of retinal pigment epithelium (RPE) alterations spanning less than two‐disc diameters. (b) Multicolor imaging and (c) corresponding *en‐face* optical coherence tomography angiography (OCT‐A) display the presence of choroidal neovascularization with polypoidal vascular dilations. (d) OCT confirms the presence of a flat irregular RPE detachment (FIPED). (e) Temporal anterior segment (AS) OCT imaging of ASSP thickness, 6 mm posteriorly to the scleral spur (SS). Note the presence of the hyporeflective lateral rectus muscle (*asterisk*) and a relatively thin ASSP as opposed to idiopathic cases. Axial length (AXL) from the displayed eye was 24.48 mm.

Scleral substantia propria, although frequently overlooked in the etiopathogenesis of CSCR, may be a critical anatomical factor influencing disease manifestation and treatment response. This major scleral tissue layer, also known as the stroma (Boote et al., 2020), has been previously defined as “the skeleton of the eye” (Trier, 2005), but it acts as a dynamic tissue with variations in relation to IOP fluctuations (Nguyen et al., 2020), ageing (Avetisov et al., 1983), ethnicity (Fazio et al., 2014) and disease. In our cohort, variations in ASSP thickness, appear to have different distributions among *simple* (59.1%) and *complex* (40.9%; Figure 3) CSCR cases (432 μm vs. 460 μm for the mean value, respectively, *p*‐value < 0.001). The two groups were uniform for age, AXL, BCVA and OCT biomarkers, however acute SRF (12% of total cases; Figure 4) was more represented in the *simple* group (94.1% vs. 5.9% cases, *p* = 0.002); conversely, persistent SRF (25.4% of total cases) was more common in the *complex* group (19.4% vs. 80.6%, *p*‐value < 0.001), which also included a higher prevalence of PCRD (11 vs. 1 case, *p*‐value < 0.001). Remarkable lower ASSP thickness values were found in case of resolved SRF (*β* = −22.3, 95% CL = −66.5/21.9; Figure 5) and steroid‐related SRF (*β* = −29.5, 95% CL = −70.9/11.9). Conversely, a greater ASSP thickness value significantly correlated with PCRD, thus potentially being a risk factor for the development of cystoid degeneration in CSCR, an OCT biomarker which was previously described as a negative predictive factor of PDT effectiveness (Nicolò et al., 2012). Among all the analysis groups, eyes with PCRD showed particularly elevated values of ASSP thickness, with a mean of 481.8 ± 43.3 μm.

Of note, ASSP measurements have not been to date investigated in clinical trials as a prognostic factor for the disease course (van Dijk et al., 2018; van Rijssen et al., 2020). In the subgroup of treated patients from our cohort (61 eyes), 63.9% had a complete response after PDT, a result which is in line with the rates obtained in the SPECTRA Trial (van Rijssen et al., 2022). In our cohort, logistic RM highlighted high ASSP thickness as a robust indicator of PDT non‐responsive patients (OR = 9.62, 95% CL = 2.44/37.9, *p* = 0.001), in contrast to the other anatomical parameters that is, BSA, age, gender and AXL.

Our results confirm the correlation between a thick sclera and *complex* CSCR cases in Caucasian patients (*β* = 26.1, 95% CL = 12.1/40.1, *p*‐value < 0.001); this finding was recently reported in a large series of Asian patients by Imanaga et al. (2024). The authors analysed 217 right eyes of 217 patients and concluded that a thick sclera may be involved in an increased area of RPE atrophy, with a higher likelihood of developing recurrent episodes and bilateral involvement. Reported mean values of scleral thickness were comparable with our results with 469.1 ± 44.4 μm for the *complex* cases (464.3 ± 46.8 in our series) and 422.2 ± 44.0 μm for the *simple* cases (429.2 ± 36.6 μm from our series). However it is important to emphasize that the prevalence and characteristics of CSCR may vary between Asian (Kido et al., 2022) and Caucasian (Kitzmann et al., 2008) cohorts: the most striking difference between our results and the report by Imanaga et al. (2024) is the different prevalence of ciliochoroidal effusion loculations in AS‐OCT of 40/217 eyes (18.4%), whereas in our cohort, we found 1/142 eyes (0.7%) with a single case occurring in *complex* CSCR (Figure 3, *arrowhead*). Similar rates (19.5%) were previously reported by Terao et al. (2022) in 164 eyes of 164 Asian patients. This aspect warrants further investigation to determine whether there are underlying biomechanical properties or different scleral outflow permeability between the two groups.

We speculate that scleral involvement in CSCR pathology may be linked to a globally reduced transscleral protein outflow alternatively or together with a strangulation effect of VVs compressed as they transit through a very thick sclera. Interestingly, a reduced transscleral albumin diffusion has been demonstrated in UES (Jackson et al., 2012). A similar process could occur in *complex* CSCR, a condition that partially overlaps with UES (Boulanger et al., 2023; Onoe et al., 2022), resulting in the accumulation of fluid in the outer choroid and in the suprachoroidal space. Increases in arterial choroidal inflow due to frank hypertension (Eom et al., 2012; Nathaniel Roybal et al., 2018) or as a result of vigorous physical activity (Cardillo Piccolino et al., 2022) could represent further negative factors for choroidal hemodynamic impairment, ultimately producing vascular congestion and a bottleneck effect at the exit site of VVs.

Our study's approach to CSCR management aligns with the evidence‐based treatment guidelines recently proposed by Feenstra et al. (2024), which advocate ICGA‐guided PDT with verteporfin as the mainstay and first‐line treatment for CSCR. However, these clinical recommendations also suggest that “CSCR with concurrent macular neovascularization should be treated with half‐dose/half‐fluence PDT and/or intravitreal injections of an anti‐vascular endothelial growth factor compound” (Feenstra et al., 2024), in our cases with vascularized FI‐PEDs we employed standalone HD‐PDT without anti‐VEGF therapy, given that this remains our current therapeutical approach in the absence of clear evidence of the usefulness of anti‐VEGF drugs in these cases. For the limited number of cases with Type 1 MNV in our cohort, this study does not allow speculations on this topic. However, it is important to note that randomized controlled clinical trials are still needed to definitively address the optimal treatment strategy for CSCR with Type 1 MNV.

Limitations of this study should be acknowledged and include the limited sample size of the treated patients (61 eyes of 56 patients), which precluded the division of the sample into a training set and verification set, and the absence of a healthy control group. Additionally, measurements of scleral thickness performed manually using AS‐OCT present novel perspectives, albeit accompanied by inherent limitations, which include the absence of automated measurement software, the inability to directly visualize the exit site of VVs and reliance on the patient's compliance regarding gaze direction. Furthermore, a secondary operator is necessitated for lid manipulation during vertical scans, involving manually holding the eyelid open to expose the sclera 6 mm posteriorly to the SS along the vertical meridian. About that point, we found no meaningful contributions coming from specific scleral sectors. This observation suggests that only nasal and temporal measurements might be used to perform ASSP thickness measures, thus avoiding the issues related to lids manipulation. However, future validation studies are warranted to support this hypothesis. Importantly, the measurement of ASSP thickness cannot be considered a representative indicator of the substantia propria thickness in the equatorial and posterior pole regions (Spaide et al., 2022). Moreover, further research is needed to validate current findings and explore underlying variations in scleral thickness in relation to age, AXL, and ocular hypotonia in CSCR patients, and the prevalence of ciliochoroidal effusion among different ethnical groups.

In conclusion, our results support the notion that CSCR may be considered a sclerochorioretinal disorder and confirm the correlation between increased ASSP thickness and *complex* CSCR (mean values: 460 μm vs. 432 μm, *p*‐value < 0.001). Moreover, increased ASSP thickness appears to be a significant anatomical predictor of incomplete response to half‐dose verteporfin PDT. These findings highlight the potential role of the scleral layer biomechanics in the pathogenesis and management of CSCR, providing insights into the disease's structural alterations in Caucasian patients.

## AUTHOR CONTRIBUTIONS

PF, JC: data collection and analysis, interpretation of the data, original writing of the manuscript, and critical revision of the manuscript. PC, RR, DM CET: data collection and interpretation. VF: supervision, statistical analysis of the results. AA: supervision, critical revision of the manuscript. FCP, ML, CME, MN: research design, interpretation of the data and critical revision of the manuscript. All authors attest that they meet the current ICMJE criteria for authorship.

## FUNDING INFORMATION

This study was supported by “Fondazione Italiana Macula ETS,” Genoa, Italy.

## ETHICAL APPROVAL

The study was approved by the cantonal commission for ethics and human research committee—Vaud (CER‐VD 2017‐00493) and was performed in agreement with the principles outlined in the Declaration of Helsinki for research involving human subjects.

## PATIENT CONSENT

Informed consent was obtained from all the patients for publication of this original article. This report does not contain any personal identifying information.
